# Expression of collagenases (matrix metalloproteinase-1, -8, -13) and tissue inhibitor of metalloproteinase-3 (TIMP-3) in naturally occurring bovine cutaneous fibropapillomas

**DOI:** 10.3389/fvets.2022.1072672

**Published:** 2023-01-12

**Authors:** Florentina Daraban Bocaneti, Gennaro Altamura, Annunziata Corteggio, Oana Irina Tanase, Mihaela Anca Dascalu, Sorin Aurelian Pasca, Ozana Hritcu, Mihai Mares, Giuseppe Borzacchiello

**Affiliations:** ^1^Department of Public Health, Faculty of Veterinary Medicine, Iasi University of Life Sciences “Ion Ionescu de la Brad”, Iaşi, Romania; ^2^Department of Veterinary Medicine and Animal Productions, University of Naples “Federico II”, Naples, Italy; ^3^Institute of Biochemistry and Cell Biology (IBBC), National Research Council (CNR), Naples, Italy; ^4^Department of Pathology, Faculty of Veterinary Medicine, Iasi University of Life Sciences “Ion Ionescu de la Brad”, Iaşi, Romania

**Keywords:** BPV, cutaneous fibropapilloma, collagenases, ECM changes, TIMP-3

## Abstract

Bovine cutaneous fibropapillomas are among the most common skin tumors in cattle; their etiology is associated with infection by bovine papillomavirus (BPV) types−1/-2 which are considered oncogenic. Degradation of the extracellular matrix (ECM), especially collagenolysis, is a key event during a series of relevant physiological processes, including tissue remodeling and repair. Various types of proteins are implicated in the regulation of ECM degradation: among these, matrix metalloproteinases (MMPs), a group of zinc-dependent endoenzymes, and tissue inhibitors of matrix metalloproteinases (TIMPs) are known to play a major role. Previous studies reported that aberrant expression of collagenolytic MMPs (MMP-1/-8/-13) and unbalancing between MMPs and TIMPs represent a critical step in tumor growth and invasion; however, studies regarding this topic in bovine cutaneous fibropapillomas are lacking. The aim of this study was to investigate the expression of the collagenases MMP-1/-8/-13 and TIMP-3 in naturally occurring fibropapillomas harboring BPV-2 DNA and normal skin samples. Here, by immunohistochemistry and western blotting analysis, we demonstrated overexpression of MMP-8/-13 along with a down-regulation of MMP-1, associated with a decrease in TIMP-3 levels in tumor compared with normal skin samples. This is the first study describing MMP-1/-8/-13 and TIMP−3 expression in bovine cutaneous fibropapillomas and our results suggest that an impaired expression of collagenases along with an imbalance between MMPs/TIMPs may contribute to an increased collagenolytic activity, which in turn could be important in ECM changes and tumors development.

## 1. Introduction

Papillomaviruses (PV) are small epitheliotropic and oncogenic DNA viruses able to infect mammals, birds, reptiles and fish as well, resulting in benign epithelial hyperproliferation and cancer (1). The Bovidae and Equidae are among the mammal species mostly affected by PVs (2). In Bovidae, cutaneous lesions associated with bovine papillomavirus (BPV) type−1 and−2 infection, classified as fibropapillomas, are well-described, being characterized by epithelium and the underlying derma proliferation and known to undergo spontaneous regression (3). In normal conditions, BPV infections are not responsible of cattle death, but under some circumstances decreased productive performances are reported and moreover, some animals are unable to defeat the infection and die to widespread cutaneous involvement (4, 5).

During tumor progression, multi-step complex events are responsible for the transformation of a healthy cell into a neoplastic cell (6), including the degradation of both basement membranes and stromal extracellular matrix by matrix metalloproteinases (MMPs) (7). MMPs, a group of zinc-dependent endoenzymes, are classified according to their modular domain structure in five families: collagenases, gelatinases, stromelysins, matrilysins, and membrane-type MMPs (8). The members of the collagenase family (MMP-1, MMP-8, and MMP-13) are known to play a crucial role in degradation of collagenous ECM through a mechanism of degrading collagens in the extracellular space, therefore MMP-mediated collagenolysis is known to be implicated in the physiological processes as well as in the progression of various pathologies (9, 10).

MMP-1 was the first collagenase described and it is expressed by fibroblasts, endothelial cells, or keratinocytes, while an increased MMP-1 expression has been reported in various inflammatory diseases and cancers (11). Interestingly, MMP-1 expression has been demonstrated in papillomavirus induced tumors such equine sarcoids (12–14).

MMP-8 (also known as collagenase 2) has collagenolytic properties; under physiological condition, MMP-8 is expressed at very low level, but in various pathological conditions, an overexpression is well-documented (15, 16).

MMP-13 (collagenase-3), originally cloned from neoplastic tissue is a much stronger collagenase than MMP-1 and MMP-8; its physiological expression is well-regulated in processes in which remodeling of collagenous extracellular matrix is required (17). However, MMP-13 seems to play multiple roles in tumor progression and metastasis since its expression is enhanced in various cancers (17, 18).

MMPs activity are controlled specifically and reversibly by a group inhibitor known as tissue inhibitors of metalloproteases (TIMPs). Unlike other TIMP variants (TIMP-1, 2, and 4), TIMP-3 has the widest inhibitory spectrum against metalloproteinases (19). Indeed, the imbalance between MMPs and their endogenous inhibitors (TIMPs) have been demonstrated to play a crucial role in the tumor microenvironment, allowing cell invasion in different cancer types such head and neck cancer, breast carcinomas, squamous cell carcinomas, or cutaneous basal cell carcinomas (20–22).

Different extracellular stimuli including growth factors and viral infection may impair MMPs and TIMPs expression (14). Accordingly, Human papillomavirus type 8 (HPV-8) has been demonstrated to stimulate the overexpression of MMP-1, MMP-8, and MMP-14, resulting in invasion of human keratinocytes (23).

Interestingly, in sarcoid, a common equine benign fibroblastic skin tumor associated to BPV-1/-2 infection, a basic mechanism responsible of cell transformation has been elucidated, including an altered expression of MMPs and TIMPs, resulting further in vicious turnover of extracellular matrix complex with excessive deposition and impaired degradation (24). Moreover, a similar impaired expression of certain MMPs and TIMPs have been recently identified in bovine cutaneous fibroapillomas [(25), manuscript submitted]; however, studies regarding this topic in bovine cutaneous fibropapillomas are scarce.

The aim of this study was to investigate the expression of the collagenases MMP-1/-8/-13 and TIMP-3 in naturally occurring fibropapillomas harboring BPV-2 and normal skin samples.

## 2. Materials and methods

### 2.1. Samples

Nineteen tumor samples (T1-T19) were obtained from cows suffering from cutaneous fibropapillomatosis. Additionally, five normal skin samples (NS = 5) were obtained from healthy bovines, slaughtered in Iasi County, Romania. The viral status of samples had been ascertained by PCR in previous studies of ours: T1-T19 resulted positive for BPV-2 but not BPV-1/-4, N1-N4 did not harbor any viral DNA, as well as N5 analyzed here by the same approach (see Table 1) (25, 26).

**Table 1:** BPV DNA presence and collagenases (MMP-1/-8/-13) and TIMP-3 protein expression in normal skin and bovine cutaneous fibropapillomas.

**Sample**	**BPV-2 DNA**	**MMP-1**		**MMP-8**		**MMP-13**		**TIMP-3**	
	**Pattern**	**Scoring**	**Pattern**	**Scoring**	**Pattern**	**Scoring**	**Pattern**	**Scoring**	
NS1	−	Cytoplasmic	++	Cytoplasmic, perinuclear	+	No	−	Cytoplasmic	++
NS2	−	Cytoplasmic	++	Cytoplasmic, perinuclear	+	No	−	Cytoplasmic	++
NS3	−	Cytoplasmic, perinuclear	++	Cytoplasmic	+	Cytoplasmic	+	Cytoplasmic	++
NS4	−	Cytoplasmic, perinuclear	+	Cytoplasmic	+	Cytoplasmic	+	Cytoplasmic	++
NS5	−	No	−	No	−	No	−	Cytoplasmic	+
T1	+	Cytoplasmic, perinuclear	+	Cytoplasmic	+	Cytoplasmic	++	Cytoplasmic	+
T2	+	Cytoplasmic, perinuclear	+	Cytoplasmic, perinuclear	++	Cytoplasmic	++	Cytoplasmic	+
T3	+	Cytoplasmic, perinuclear	++	Cytoplasmic, perinuclear	+	Cytoplasmic	++	Cytoplasmic	++
T4	+	Cytoplasmic, perinuclear	+	Cytoplasmic, perinuclear	++	Cytoplasmic	+	Cytoplasmic	+
T5	+	Cytoplasmic, perinuclear	+	Cytoplasmic, perinuclear	++	Cytoplasmic	++	Cytoplasmic	+
T6	+	Cytoplasmic	+	Cytoplasmic, perinuclear	++	Cytoplasmic	+	Cytoplasmic, perinuclear	+++
T7	+	Cytoplasmic, perinuclear	+	Cytoplasmic, perinuclear	++	Cytoplasmic	+++	Cytoplasmic, perinuclear	++
T8	+	Cytoplasmic, perinuclear	+	Cytoplasmic, perinuclear	++	Cytoplasmic	++	Cytoplasmic	++
T9	+	Perinuclear	+	Cytoplasmic, perinuclear	++	Cytoplasmic	+	Cytoplasmic	++
T10	+	No	−	Cytoplasmic	+	Cytoplasmic	++	Cytoplasmic, perinuclear	++
T11	+	Cytoplasmic	+	Cytoplasmic, perinuclear	++	Cytoplasmic	++	No	−
T12	+	No	−	Cytoplasmic	+	Cytoplasmic	+	Cytoplasmic	+
T13	+	Cytoplasmic, perinuclear	+	Cytoplasmic, perinuclear	++	Cytoplasmic	++	Cytoplasmic, perinuclear	++
T14	+	No	−	Cytoplasmic, perinuclear	++	Cytoplasmic	+	Cytoplasmic	++
T15	+	Cytoplasmic, perinuclear	++	Cytoplasmic, perinuclear	+	Cytoplasmic	+++	Cytoplasmic	+
T16	+	No	−	No	−	No	−	Cytoplasmic	+
T17	+	Cytoplasmic, perinuclear	+	Cytoplasmic, perinuclear	++	Cytoplasmic	+++	Cytoplasmic	+
T18	+	Cytoplasmic, perinuclear	−	Cytoplasmic, perinuclear	+	Cytoplasmic	++	Cytoplasmic	+
T19	+	Cytoplasmic, perinuclear	+	Cytoplasmic, perinuclear	++	Cytoplasmic	++	Cytoplasmic	+

–, negative; +, weak, individual positive cells; ++, foci of moderate positivity; +++, >50% of cells moderately to strongly positive.

### 2.2. Immunohistochemistry

Paraffin sections of 19 fibropapillomas and five normal skins underwent the immunohistochemical protocol (streptavidin biotin- peroxidase method—Novolink Polymer Detection System; Leica Biosystems, NewCastle, United Kingdom) as described by the authors in a previous study (25). The antibodies anti-MMP-1 (3B6: sc-21731, Santa Cruz), anti-MMP-8 (B-1: sc-514803, Santa Scruz), anti-MMP-13 (MA5-14238, Thermofisher), and anti-TIMP-3 (B-2: sc-373839, Santa Cruz) were applied for 1 h at room temperature (RT). The treatment with diaminobenzidine was used to visualize the specific immunoreactivity. The immunolabelling procedure included canine mammary carcinoma samples as positive control (27) and negative control sections, where primary antibodies were omitted and replaced with phosphate buffer saline instead. MMPs and TIMPs immunoreactivity were scored as previously described by Bocaneti et al. (28): –, negative; +, weak, individual positive cells; ++, foci of moderate positivity; +++, > 50% of cells moderately to strongly positive. The immunoreactivity was scored by two observers (FDB and GB) under blinded conditions.

### 2.3. Western blotting

Biochemical analysis was performed on four normal skin samples and nine fibropapilloma samples. Protein extraction, electrophoresis and blotting were performed as previously described (25). The membranes were blocked with 5% bovine serum albumin (BSA) in TBS-0.1% Tween buffer (10 mM Tris–HCl, pH 7.4, 165 mM NaCl, 0.1% Tween) at RT, washed with TBS-0.1% Tween, and incubated with the following primary antibodies: anti-MMP-1 (1:1000), anti-MMP-8 (1:500), anti-MMP-13 (1:1000) and anti-TIMP-3 (1:500), respectively. After appropriate washing steps in TBS-0.1% Tween buffer, goat anti-mouse (GE Healthcare #LNA931V/AH) and donkey anti-rabbit (GE Healthcare #LNA934V/AH) secondary antibodies conjugated with horseradish peroxidase were applied for 1 h at RT, and protein bands were visualized by enhanced chemiluminescence (ECL, Bio-Rad) at ChemiDoc gel scanner (Bio-Rad). To ensure comparable amounts of proteins for each sample, the blots were stripped and reprobed against mouse anti-β-actin antibody (C-2: sc-8432, Santa Cruz) at 1:500 dilution. Densitometric analysis for protein quantization was achieved by using Image Lab software (Bio-Rad). The protein concentrations were normalized to the β-actin levels and expressed as the densitometric ratio.

### 2.4. Statistical analysis

For statistical analysis, Student's *t*-test was performed using SPSS 17.0 software (SPSS Inc.), and differences were considered to be statistically significant for ^*^P < 0.05 or ^**^P < 0.01.

## 3. Results

### 3.1. Immunohistochemistry

The individual expression patterns and the scoring for MMP-1, MMP-8, MMP-13, and TIMP-3 in five normal skin samples and 19 bovines cutaneous fibropapillomas are detailed in Table 1.

**Figure 1:**
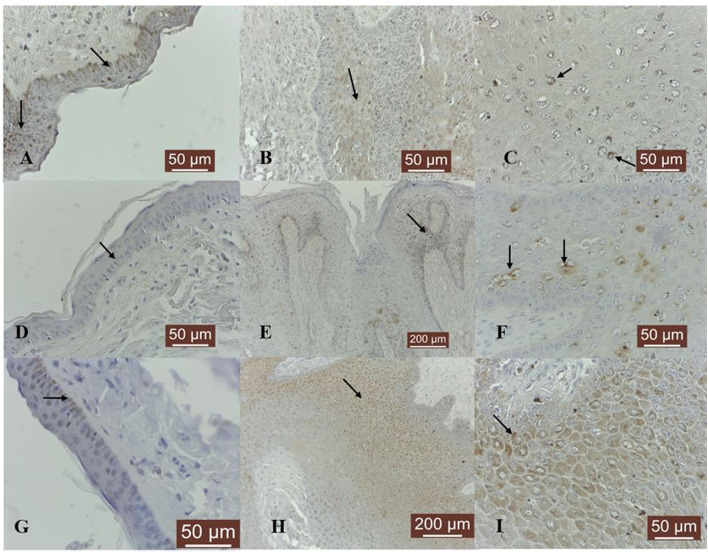
MMP-1/-8/-13 immunostaining in bovine normal skin and fibropapillomas: **(A)** In bovine normal skin, a moderate immunoreactivity for MMP-1 is noted in the cytoplasm of epithelial cells (black arrows); 40 X. **(B)** In bovine fibropapillomas MMP-1 is expressed as a weak cytoplasmic (black arrow) and perinuclear pattern both in epithelial and fibroblast cells; 20 X. **(C)** MMP-1 in fibropapilloma: note the fine granular perinuclear positivity of upper epithelial cells (black arrow); 40 X. **(D)** In normal skin samples, MMP-8 showed a specific weak immunoreactivity in a cytoplasmic (black arrow) pattern in all epithelial cells; 40 X. **(E)** In fibropapillomas, MMP-8 was strongly expressed in epithelial cells, while the fibroblasts showed a weak reactivity; 20 X. **(F)** Note a detailed fine strong granular cytoplasmic and perinuclear (black arrow) pattern in scattered epithelial cells from spinosum layer; 40 X. **(G)** Normal skin: MMP-13 weak cytoplasmic immunoreactivity (black arrow) predominantly in basal cell layer; 40 X. **(H)** Fibropapillomas: MMP-13 appeared as a finely granular cytoplasmatic staining in epithelial cells (black arrow); 20 X. **(I)** A detailed MMP-13 moderate cytoplasmic (black arrow) immunosignaling confined to all epithelial cells; 40 X. Streptavidin-biotin-peroxidase staining.

We evaluated the expression of MMP-1, known to plays a significant role in the degradation of different types of collagens in extracellular matrix remodeling. MMP-1 was expressed in four out of five normal skin samples as a moderate granular cytoplasmic and perinuclear pattern. The staining was recorded predominantly in the basal cell layer (Figure 1A).

MMP-1 expression was recorded in 14 out of 19 samples. The immunoreactivity in fibropapillomas was confined to upper epithelial layers, where a cytoplasmic and perinuclear localization was detected (Figure 1B); furthermore, some tumor samples were characterized by the presence of spinous cells with a perinuclear strong granular pattern (Figure 1C), while the cells from the basal layer were negative; moreover, in 2/19 (11%) samples, a moderate labeling was recorded in cytoplasm of fibroblast cells (Figure 1B).

Collagenase MMP-8 was expressed in four out of five normal skin samples, exhibiting predominantly a weak cytoplasmic and perinuclear immunosignal in basal and parabasal layers (Figure 1D). Eighteen out of 19 (95%) fibropapilloma samples showed specific cytoplasmic and perinuclear immunoreactivity (Figure 1E) mainly in the basal epithelial layer, with scattered positive cell found in the spinosum layer, showing a pronounced perinuclear staining (Figure 1F); a weak positivity was recorded in six out of 19 (32%) samples and a moderate intensity was noted in 12 out of 19 samples (63%). Four out of 19 (21%) tumor samples showed a weak cytoplasm expression in fibroblasts (Figure 1E).

MMP-13 expression was detected as weak cytoplasmic immunosignal in basal layer in two out of five normal skin samples (Figure 1G). Fibropapillomas featured a weak positivity in 5/19 (26%) tumor samples, moderate in 10/19 (53%), and strong in 3/19 samples (16%), characterized by a finely granular pattern in the cytoplasm mostly in the upper epithelial layers (Figure 1H), while the basal cells displayed a weaker expression (Figure 1I).

**Figure 2:**
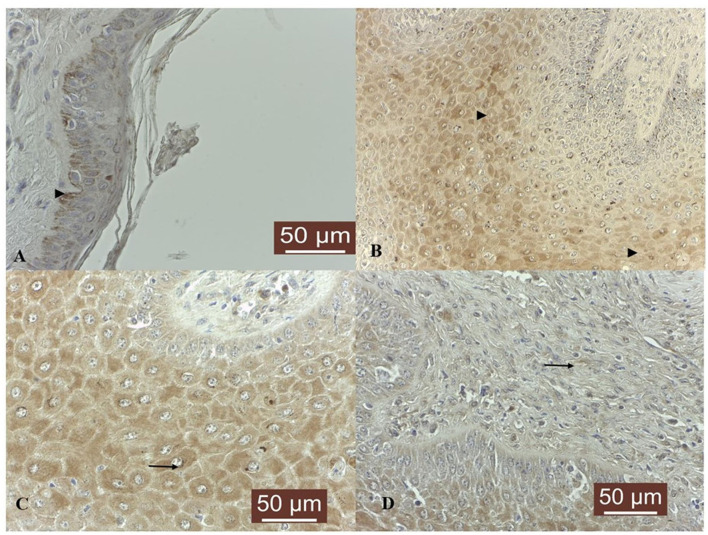
TIMP-3 immunoexpression in bovine normal skin and fibropapillomas: **(A)** TIMP-3 is detected as moderate cytoplasmic (black arrow) pattern in normal skin basal cell layer; 40 X. **(B)** In tumor samples, TIMP-3 expression was recorded as a weak to moderate finely granular staining pattern in the cytoplasm of upper epithelial cells (black arrowheads); 20 X. **(C)** Note a detailed TIMP-3 moderate granular cytoplasmic and perinuclear pattern (black arrow) in spinous layer; 40 X. **(D)** Fibroblasts showed a weak cytoplasm and perinuclear immunoreactivity (black arrow); 40 X. Streptavidin-biotin-peroxidase staining.

In five out of five normal skin samples, a moderate cytoplasmic TIMP-3 immunoreactivity was noted in basal cells and some cells from upper layers showed weak positivity (Figure 2A). TIMP-3 expression was weak in 10 out of 19 (53%), moderate in 7/19 (37%), and strong in 1/19 (5%; sample T6) fibropapillomas, where a finely granular staining pattern was recorded in the cytoplasm of epithelial cells layer (Figure 2B); in some samples (32%) a similar cytoplasmic immunoreactivity pattern was seen in fibroblasts (Figures 2C, D). Additionally, few cell from spinous layer, along with fibroblast cells from the dermal layer, displayed an evident perinuclear immunostaining (sample T6; Figure 2C).

### 3.2. Western blot analysis

**Figure 3:**
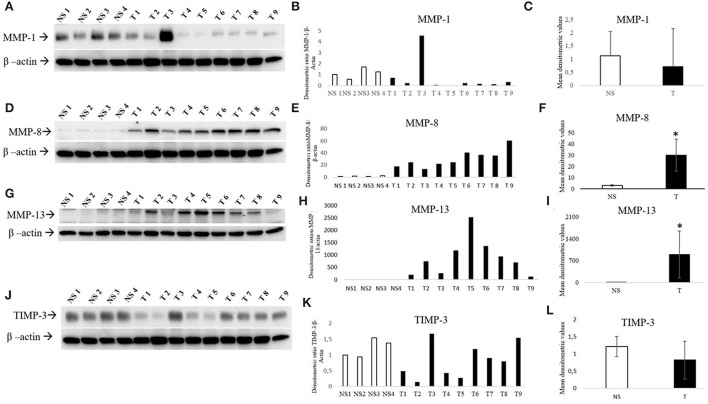
Western blotting analysis of MMP-1/-8/-13 and TIMP-3 in bovine normal skin (NS) and fibropapillomas (T). Western blotting and densitometric measurements of MMP-1/-8/-13 and TIMP-3 in NS1-NS4 and T1-T9 samples **(A–L)**. Representative gels showing an enhanced amount of MMP-8/-13 **(D, G)** and decreased expression of MMP-1 **(A)** and TIMP-3 **(J)** in tumor compared with normal skin samples are shown. The blots were stripped and reprobed with anti- β-actin antibody to confirm comparable loading of proteins in each lane and allow normalization. **(B, E, H, K)** Individual densitometric values of MMP-1/-8/-13/ and TIMP-3 for each sample expressed as densitometric ratio with β-actin. **(C, F, I, L)** Mean densitometric values +/- standard deviations for MMP-1/-8/-13 and TIMP-3 in NS vs. T groups (*: statistically significant by *t*-test, *P* < 0.05).

Four normal skin samples and nine bovine fibropapillomas samples were subjected to Western blotting analysis. The anti-MMP-1, anti-MMP-8, anti-MMP-13, and anti-TIMP-3 antibodies yielded a band of expected molecular weight, confirming the specificity of immunohistochemical staining. MMP-1 appeared down-regulated in most of tumor specimens (8/9, 89%) when compared to normal skin samples, except in sample T3 (Figure 3). Densitometric analysis of individual samples as well as mean tumor vs. normal skin values confirmed the results, although the difference between the two sample groups was not statistically significant mostly due to sample T3. Further, MMP-8 was over-expressed in all tumor samples (Figure 3D), as confirmed also by individual (Figure 3E) and mean densitometric values (Figure 3F) of tumor vs. normal skin samples, and the difference between the two groups was statistically significant (*t*-test; *P* < 0.05).

Collagenase MMP-13 expression was low to undetectable in normal skin samples, whilst in 9/9 tumor samples an overexpression (Figure 3G) was evident and confirmed by densitometric analysis (Figure 3H). Moreover, the mean densitometric values (Figure 3I) revealed a statistically significant difference between normal and tumor samples (*t*-test; ^*^P < 0.05).

When compared to normal skin samples, TIMP-3 appeared down-regulated in most of tumor samples (Figure 3J). Although the difference between samples groups was not statistically significant, the densitometric analysis of individual samples (Figure 3K) as well as mean tumor (Figure 3L) vs. normal skin values confirmed the trend of results.

## 4. Discussions

Cutaneous fibropapillomas, known as benign proliferative lesions commonly diagnosed in bovine, are characterized by hyperproliferation of epithelial cells and dermal fibroblasts and induced by well-known oncogenic BPV type−1/-2 (29). Furthermore, BPV-1/-2 are considered to be the causative agents of equine sarcoid (30) and it has been hypothesized by Martano et al. (24) that the basic mechanism for the development of equine sarcoids could be an imbalance of ECM deposition and degradation as seen also during pathologic wound healing, which in turn is supported by altered expression levels between MMPs and their inhibitors (TIMPs). Indeed, both *in vivo* and *in vitro* studies have shown that the over-expression and activation of MMPs is induced by BPV (12–14, 24). Moreover, in line with our recently work which report an imbalance between MMPs and TIMPs expression and activity in presence of BPV-2 in bovine cutaneous fibropapilloma (25), in this current study we demonstrate for the first time, expression of collagenases (MMP-1, MMP-8, and MMP-13) and TIMP-3 in bovine cutaneous fibropapillomas associated with BPV-2 infection.

During tumor development multi-step processes are described, involving degradation of structural barriers such as basement membrane and collagenous extracellular matrix and migration of cells through the degraded matrix (31). In particular, the overexpression and increased levels of collagenolytic matrix metalloproteinases (MMP-1,−13, and−8) have been shown to be associated with progression of certain tumors, therefore their individual or concerted action is required to initiate collagenolysis (32).

The data regarding MMP-1 expression in BPV-induced tumors are contradictory. In a study on equines sarcoids, a moderate MMP-1 expression was demonstrated in both neoplastic fibroblasts and epithelial cells, particularly in epidermal keratinocytes concomitantly with a moderately to strongly expression in epithelial cells of normal skin (12). Although in our study we observed an overexpression of MMP-1 in 1/9 fibropapillomas by biochemical analysis, our results are in contrast with those reported by Yuan et al. (14), who suggested that MMP-1 overexpression is essential for the transformation of sarcoid fibroblasts in a BPV-1 dependent manner and moreover BPV-1 oncoproteins E5, E6, and E7 are contributing to increased MMP-1 expression, thus concurring to cellular transformation (13, 14). In this context, it is interesting to mention that in bovine cutaneous fibropapillomas, we demonstrated by immunohistochemistry a weak to moderate cytoplasmic and perinuclear collagenase MMP-1 expression, comparing to normal skin samples where a moderate reaction was seen; these differences in expression normal vs. tumors were confirmed biochemically, suggesting a potential contribution by potentiating the collagenolysis process. However, a recent study on *in vivo* and *in vitro* models designed to explore molecular nature of equine sarcoids indicate that BPV-1 infection contributes to diminished MMP-1 expression level (33), results which are consistent with our study. On the other hand, in knockdown MMP-1 cervical cancer cell lines it has been demonstrated a reduced invasion ability compared to the control, suggesting that knockdown of MMP-1 is responsible of reduced proliferation, invasion and migration (34). Taking into consideration all these data, we may suggest that in bovine cutaneous fibropapillomas, MMP-1 low expression may be responsible of tumor development and migration ability.

It is noteworthy that MMP-8, previously thought to be present only in neutrophils, has been later demonstrated to be expressed in various cancers such melanoma and head-and-neck carcinoma (35). Interestingly, when MMP-8 enzyme was analyzed, we revealed for the first time predominantly a moderate cytoplasmic and perinuclear immunoreactivity, whilst the overexpression in tumor sample comparing to normal skin tissue was confirmed with western blot analysis. This data is consistent with previous findings in human basal cell carcinoma and squamous cell carcinomas (16, 32), where overexpression was reported and further associated with keratinocyte invasiveness. Thus, taking into consideration these data, we may speculate that in BPV-2 positive fibropapillomas, overexpression of MMP-8 may interfere with tumor further development.

Given its powerful and destructive action toward ECM, it is not surprising that expression of MMP-13 in tumor creates a more favorable environment for tumor growth, whilst its overexpression is often associated with tumor aggressiveness and poor prognosis (36). Indeed, expression of MMP-13 had been reported in various tumors including colorectal, prostate, esophageal, breast and head, and neck cancer (HNSCC) (17, 36). Consistently, our data demonstrated that MMP-13 is expressed in all the epithelial layers in almost all analyzed cutaneous fibropapillomas and generally over-expressed with respect to normal skin, suggesting an involvement of this collagenase in bovine epithelial neoplastic transformation.

TIMP-3 is secreted by most cell types and is sequestered at the cell surface, where it is bound by components of the ECM, while its main function is consisting in tumor suppression (37). Reduced protein expression of TIMP-3 was associated with reduced tumor differentiation and increased metastatic activity, since its binding to MMPs in tumor cells is leading to a reduced ECM-degrading activity (38). Moreover, a decreased expression of TIMP-3 has been reported in cervical intraepithelial neoplasia and cervical cancer related to HPV, which in turn may be responsible implicitly by an impaired extracellular matrix degradation activity and cyto-architectural abnormalities (39). In our study, in fibropapillomas, a weak to moderate expression was noted by immunohistochemistry mostly in the upper epithelial layers, and next, by western blot analysis, a down-regulated expression was confirmed, which may support an important contribution in impairing the collagenases activity.

## 5. Conclusions

This is the first study describing MMP-1/-8/-13 and TIMP−3 expression in bovine cutaneous fibropapillomas and our results suggest that an impaired expression of collagenases together with an imbalance between MMPs/TIMPs may contribute to an increased collagenolytic activity, which in turn could be important in ECM changes and tumors development.

## Data availability statement

The original contributions presented in the study are included in the article/supplementary material, further inquiries can be directed to the corresponding author.

## Ethics statement

The animal study was reviewed and approved by Ethics and Deontology Committee, Iasi University of Life Sciences “Ion Ionescu de la Brad”, Iaşi, Romania.

## Author contributions

FDB, OIT, MAD, OH, and SAP performed IHC. FDB and GA performed WB and densitometric analysis. FDB, GA, AC, MM, and GB drafted the manuscript. All authors contributed to the article and approved the submitted version.
